# Supplementary material for: Correlation between quality of life of cardiac patients and caregiver burden
Source: PLoS One. 2020 Aug 3;15(8):e0237099. doi: 10.1371/journal.pone.0237099 (PMC7398537; doi:10.1371/journal.pone.0237099)
Supplement: Attachment — Submitted filename: Review_PLOS one_2020.pdf [file pone.0237099.s001.pdf]

Comments to Author:

Ref. No.: PONE-D-20-10353

The work by Subih et al. entitled " *Quality of life of Cardiac patients as a predictor of Caregivers' Burden*" focuses on the association between caregivers burden and the quality of life of the cardiac patients they care for – investigating the caring of carers. The authors report that lower age, shorter education and higher quality of life of the patients were all statistically significant predictors of the caregivers burden. The objective of the study is interesting, the manuscript is engaging and the methodology is broadly appropriate (which is often challenging within studies focusing on Quality of Life). However, I have some concern associated with the structure on the manuscript, the language used and reporting of data listed below.

MAJOR

1. For a broader audience in a journal as PLOS One please specify concepts and the setting early in the introduction, as they are somewhat unknown for the reader. I suggest a re-structuring of the introduction. E.g. move page 3 line 53-55 up – as it contains key background information.
2. I find some of the description regarding the method section too detailed while the description of some important methods are not adequately described.
3. The study assessed 200 caregivers and 203 patients, however the response rate around 80%. There are some figures e.g. in table 1 that do not quite align with this. E.g. the title n=200, but you previously stated a response rate of 80%? Furthermore, the population (in this cross-sectional design) stems from "one educational, two public, and three private hospital clinics in Jordan". With the inherent limitations of a cross-sectional design regarding selection bias and generalizability I would suggest a more rigorous description of how the population was chosen.
4. In the conclusion you state that "*Therefore, we recommend that future interventional studies to test such training programmes evaluate their efficiency levels in terms of outcomes for both patients and their caregivers in the long term*." but the objective of the study was to "*explore the predictors of caregivers' burden.*" I suggest a more stringent focus on concluding on the findings of the study. Are there evidence in current training programs?
5. The data is interesting, however their presentation needs adjustment. According to table 2-4 I would appreciate if you reported the DOBI and QLI-Cardiac 4 findings in context with the demographic variables. E.g. is there a relationship between the total DOBI scale and the time spent with the patient? Would it be more appropriate to add a figure for this purpose? It would be very illustrative with a forest plot.
6. The study assesses the caregivers burden by the DOBI in a Arabic version. Please reference that this translated version is validated. I am not aware, whether the approach with submitting the instrument to local faculty members for approval is a standardized method. Please clarify.

7. Please discuss on the finding that the caregiving burden increases when the patients HRQoL increases. A potential cause?
8. The title of the manuscript is not completely aligned with the conclusion in the abstract. I would suggest adjusting.
9. Please consider grammatical assistance or make adjustments in the language throughout.
10. In the abstract conclusion you describe that the burden "is related to some modifiable factors" and state that the variables that are of significance are age, years of educational level and quality of life of the patients. Age is hardly modifiable, years of educational level might be, however you find that increased quality of life of the patients is associated with a higher burden. Its seems counterintuitive to modify the latter, please elaborate.

MINOR

1. Do you have more data on the socioeconomic position? It would be interesting to know and potentially be a relevant confounding variable regarding economy?
2. Have you gathered data on the quality of the relationship between caregiver and the patient? Literature suggests that this is highly relevant.
3. Why was a cardiac population chosen? Please clarify.
4. In the Health-related quality of life is mentioned whereas Quality of Life is used without. Please elaborate on your definition on the difference.
5. Please detail the data analysis section on the descriptive statistics.
6. Please specify which correlation tests were used in the data analysis section.
7. Page 2 line 35: "Improving patient´s outcomes is now the focus?
8. Page 2 linje 40: You have just abbreviated HRQoL, please use abbreviation.
9. Please reference statement "The health-related quality of life for cardiac patients and the burden of care to the caregivers is core for healthcare planning, especially when no previous or limited experience in these conditions exists."
10. Page 3: Line 43-45: Please re-write as the current version comes across as obvious.
11. Page 3 line 45: Use a different wording than "somewhat mandatory". Please specify.
12. Page 3 line 54. Is the use of family members as providers of care to patients unique for the Arab and Islamic culture or similar across geography and cultures? Please specify for the broad audience. Please put the findings into a broader context e.g. western culture.
13. Page 4 line 70: "Characteristics of those caregivers". There seems to be a missing a part of the sentence? "those caregivers that…."?
14. Page 4 line 71 "…chilvarous…" I suggest finding a more appropriate academic wording? Although I agree on the purpose of the wording.
15. Page 4 line 82: Please specify the statistical software G power, by version used and country of origin.
16. The study uses a convenience sample as population. Please add this to the limitations section discussing potential sampling error and lack of achieving a representative population.
17. Page 4 line 91: "…would they like to take part in the research" please rephrase.
18. Page 5 line 94-95. "with the option to withdraw at any time, even if they had already started to participate". Please erase second part of the sentence.
19. Page 5 line 97 "of our university" Please write the specific university.

20. Page 6 line 135 “Two hundred caregivers completed the questionnaire (response rate of 80%).” It is not clear to me what the actual number is based on this sentence. Was it two hundred completed (please use actual numbers) or 200*0,8?
21. During the results section please use mean/ median values rather than “average”.
22. Page 7 line 142. Please specify what you mean by a *diagnosis* of stent?
23. Please erase sentence “Mean scores on the DOBI…” as this is correctly states in the method section
24. I suggest beginning the discussion with stating your main findings.
25. Page 10 Line 214-215 “*When love and beliefs synergise to cover the act of giving with the umbrealla of the blessing of a higher power, some psychological factors may be modified*” Please add reference for academic rigorous.
26. Page 10 line 237 Please use different verb than conduct in relation to generalization.
27. In table 1. Please specify what “other” entail.
28. In table 1. I suggest using n (%) instead of % (n) version.
29. In table 1 you find that the mean time spent in caring is 6,9 hours, which seems remarkably high if it is direct contact hours with the patient (around 2/3 of the awake hours of the caregiver). Please clarify on this.
30. In table 1. You state “Type of condition” as a variable. I would term “HF” a condition, but not necessarily the remaining. PTCA is not a condition. Post-surgery is on the other hand. Please clarify your choice.
31. In table 1. Add abbreviation to CABG.
32. In table 4. Please report all the demographic variables and their association to the caregivers burden instead of solely those of statistical significance.
33. Table 4. I would suggest a more standardized format of presenting your findings.
34. It is not stated whether the study used complete case analysis or imputed for missing data – and if which statistical imputation was used.
35. I would suggest reporting mean scores with SD instead of just means.
36. The terms prediction and association seems to be loosely used, however I suggest the usage of association instead of prediction throughout
37. Remember to include the strengths of the study and not only report your limitations.
38. In introduction it is stated “Improving patients’ outcomes is now the focus of national and international healthcare plans, especially among nurse practitioners (2)”, however your referenced article is from 20012. Have no new articles on this matter been published?
