# Supplementary material for: Correlation between quality of life of cardiac patients and caregiver burden
Source: PLoS One. 2020 Aug 3;15(8):e0237099. doi: 10.1371/journal.pone.0237099 (PMC7398537; doi:10.1371/journal.pone.0237099)
Supplement: Attachment — Submitted filename: Response to Reviewers.docx [file pone.0237099.s002.docx]

**Response to Reviewers**

|  | **Reviewer comments** | **response** |
| --- | --- | --- |
| **Major** | | |
| 1- | For a broader audience in a journal as PLOS One please specify concepts and the setting early in the introduction, as they are somewhat unknown for the reader. I suggest a re-structuring of the introduction. E.g. move page 3 line 53-55 up – as it contains key background information. | Concepts as QoL and caregivers and caregivers’ burden were defined in the introduction. Restructuring of introduction paragraph had been done. |
| 2- | I find some of the description regarding the method section too detailed while the description of some important methods are not adequately described | It is not clear which section are too detailed and which are not described adequately however the manuscript had been rewritten and modified. |
| 3- | The study assessed 200 caregivers and 203 patients, however the response rate around 80%. There are some figures e.g. in table 1 that do not quite align with this. E.g. the title n=200, but you previously stated a response rate of 80%? Furthermore, the population (in this cross-sectional design) stems from “one educational, two public, and three private hospital clinics in Jordan”. With the inherent limitations of a cross-sectional design regarding selection bias and generalizability I would suggest a more rigorous description of how the population was chosen. | The sample size corrected it is 200 for both caregivers and cardiac patients. Response rate had been clarified more as requested (250 questionnaire distributed but 200 completed (so 200/250*100= 80% response rate).  Sample was chosen by convenience method for those had the eligible criteria and agree to participate from the hospitals that agree to collect data from their clinics. |
| 4- | In the conclusion you state that “Therefore, we recommend that future interventional studies to test such training programmes evaluate their efficiency levels in terms of outcomes for both patients and their caregivers in the long term.” but the objective of the study was to “explore the predictors of caregivers´ burden.” I suggest a more stringent focus on concluding on the findings of the study. Are there evidence in current training programs? | Conclusion had been changed. |
| 5- | The data is interesting, however their presentation needs adjustment. According to table 2-4 I would appreciate if you reported the DOBI and QLI-Cardiac 4 findings in context with the demographic variables. E.g. is there a relationship between the total DOBI scale and the time spent with the patient? Would it be more appropriate to add a figure for this purpose? It would be very illustrative with a forest plot. | Table (4) added that showed Relationship between caregivers’ characteristics and their burden (DOBI) while QLI-version 4 will be published in another study.  Time spent in caring with cardiac patients was not correlated with level of burden. |
| 6- | The study assesses the caregivers burden by the DOBI in a Arabic version. Please reference that this translated version is validated. I am not aware, whether the approach with submitting the instrument to local faculty members for approval is a standardized method. Please clarify. | The tool is valid by using content validity and calculate content validity index CVI which was 0.8. Faculty members who we ask them to check tool validity are clinical nurse specialist with clinical experience, this method as Polit & Beck (2017) book mentioned is acceptable. This had been clarified more in the manuscript as requested. |
| 7- | Please discuss on the finding that the caregiving burden increases when the patients HRQoL increases. A potential cause? | This findings had been elaborated in the discussion section as requested |
| 8- | The title of the manuscript is not completely aligned with the conclusion in the abstract. I would suggest adjusting. | Conclusion changed to fit with findings. |
| 9- | Please consider grammatical assistance or make adjustments in the language throughout. | Professional language company had adjust the whole manuscript (accoedk@gmail.com). |
| 10- | In the abstract conclusion you describe that the burden “is related to some modifiable factors” and state that the variables that are of significance are age, years of educational level and quality of life of the patients. Age is hardly modifiable, years of educational level might be, however you find that increased quality of life of the patients is associated with a higher burden. Its seem counterintuitive to modify the latter, please elaborate. | Conclusion changed |

|  | **Reviewer comments** | **response** |
| --- | --- | --- |
| **Minor** | | |
| 1- | Do you have more data on the socioeconomic position? It would be interesting to know and potentially be a relevant confounding variable regarding economy? | No, we did not include this variable, usually Jordanian population is sensitive toward such variables |
| 2- | Have you gathered data on the quality of the relationship between caregiver and the patient? Literature suggests that this is highly relevant. | No, but we recommend to add this variable in future studies |
| 3- | Why was a cardiac population chosen? Please clarify. | We added a paragraph at the beginning of introduction section that clarify why we choose cardiac patients in which statistics showed that CVD are cause no one in death in Jordan and globally |
| 4- | In the Health-related quality of life is mentioned whereas Quality of Life is used without. Please elaborate on your definition on the difference. | Thanks for letting us notice this and a correction in the manuscript applied in which we consistently use quality of life. This is because QoL is broader than HRQoL and the tool we use measure QoL in general with all its aspects, and this tool widely used to assess QoL of cardiac patients. |
| 5- | Please detail the data analysis section on the descriptive statistics. | Details added |
| 6- | Please specify which correlation tests were used in the data analysis section. | Correlation tests had been specified as requested |
| 7- | Page 2 line 35: “Improving patient´s outcomes is now the focus? | This sentence although it was according to a reference but it had been removed when we modify all the manuscript. |
| 8- | Page 2 line 40: You have just abbreviated HRQoL, please use abbreviation. | All abbreviations had been corrected |
| 9- | Please reference statement “The health-related quality of life for cardiac patients and the burden of care to the caregivers is core for healthcare planning, especially when no previous or limited experience in these conditions exists.” | After many modifications in manuscript this sentence had been removed |
| 10- | Page 3: Line 43-45: Please re-write as the current version comes across as obvious. | We rewrite it as  “Cardiac patients suffer from frequent rehospitalization either due to the disease progress or its complications during management (7). Therefore, it has become necessary to direct attention and care to at-home caregivers, such as partners, sons, daughters, relatives, or others (8).” |
| 11- | . Page 3 line 45: Use a different wording than “somewhat mandatory”. Please specify. | Changed to necessary |
| 12- | Page 3 line 54. Is the use of family members as providers of care to patients unique for the Arab and Islamic culture or similar across geography and cultures? Please specify for the broad audience. Please put the findings into a broader context e.g. western culture. | The paragraph had been modified for broader audience as requested. |
| 13- | Page 4 line 70: “Characteristics of those caregivers”. There seems to be a missing a part of the sentence? “those caregivers that….”? | Paragraph is modified” Additionally, there is a gap in the cardiac literature regarding the characteristics of those caregivers, which when recognized, will be useful for designing targeted training and support interventions that will guide this virtuous population gently and effectively through the whole care process”. |
| 14- | Page 4 line 71 “…chilvarous…” I suggest finding a more appropriate academic wording? Although I agree on the purpose of the wording. | Changed to virtuous |
| 15- | Page 4 line 82: Please specify the statistical software G power, by version used and country of origin. | Had been specified  Version 3.0.10 Germany |
| 16- | The study uses a convenience sample as population. Please add this to the limitations section discussing potential sampling error and lack of achieving a representative population. | Had been added thanks for your note |
| 17- | Page 4 line 91: “…would they like to take part in the research” please rephrase. | Changed” whether they would like to take part in the research” |
| 18- | Page 5 line 94-95. “with the option to withdraw at any time, even if they had already started to participate”. Please erase second part of the sentence. | erased |
| 19- | Page 5 line 97 “of our university” Please write the specific university. | Had been specified “Ethical approvals were obtained from the Institute Review Boards of both Al Zaytoonah University and the corresponding hospitals.” |
| 20- | Page 6 line 135 “Two hundred caregivers completed the questionnaire (response rate of 80%).” It is not clear to me what the actual number is based on this sentence. Was it two hundred completed (please use actual numbers) or 200*0,8? | True, that’s why we rewrite it in sample &setting  “A convenience sample of a total of 200 caregivers and 200 patients with cardiac diseases agreed to participate (out of 250 questionnaire distributed so response rate 80 %).”  200/250*100= 80% |
| 21- | During the results section please use mean/ median values rather than “average”. | Note had been taken in consideration and changed to mean. |
| 22- | Page 7 line 142. Please specify what you mean by a diagnosis of stent? | It had been changed to PCI: Percutaneous Coronary Interventions which is more scientific and specific |
| 23- | Please erase sentence “Mean scores on the DOBI…” as this is correctly states in the method section | erased |
| 24- | I suggest beginning the discussion with stating your main findings. | Suggestion had been taken and findings were added at the beginning of each section of discussion. |
| 25- | Page 10 Line 214-215 “When love and beliefs synergise to cover the act of giving with the umbrealla of the blessing of a higher power, some psychological factors may be modified” Please add reference for academic rigorous. | This sentence erased |
| 26- | Page 10 line 237 Please use different verb than conduct in relation to generalization. | Changed “is limited” |
| 27- | In table 1. Please specify what “other” entail. | It specified at the end of the table, which include parents, friends, neighbors, and relatives |
| 28- | In table 1. I suggest using n (%) instead of % (n) version. | Changed as requested |
| 29- | In table 1 you find that the mean time spent in caring is 6,9 hours, which seems remarkably high if it is direct contact hours with the patient (around 2/3 of the awake hours of the caregiver). Please clarify on this. | It include direct and indirect time spent in caring. Added at the end of table (1). |
| 30- | In table 1. You state “Type of condition” as a variable. I would term “HF” a condition, but not necessarily the remaining. PTCA is not a condition. Post-surgery is on the other hand. Please clarify your choice. | Changed to diagnosis |
| 31- | In table 1. Add abbreviation to CABG | Abbreviation was added at the end of table 1 |
| 32- | In table 4. Please report all the demographic variables and their association to the caregiver’s burden instead of solely those of statistical significance. | All had been added |
| 33- | Table 4. I would suggest a more standardized format of presenting your findings. | Format changed |
| 34- | It is not stated whether the study used complete case analysis or imputed for missing data – and if which statistical imputation was used. | In our data we found just few missing data that did not have major effect on the results so no need for statistical imputation. |
| 35- | I would suggest reporting mean scores with SD instead of just means | Done as requested |
| 36- | The terms prediction and association seems to be loosely used, however I suggest the usage of association instead of prediction throughout | had been modified and rewritten. |
| 37- | Remember to include the strengths of the study and not only report your limitations. | Strength points had been added as well as limitations |
| 38- | In introduction it is stated “Improving patients’ outcomes is now the focus of national and international healthcare plans, especially among nurse practitioners (2)”, however your referenced article is from 20012. Have no new articles on this matter been published? | Sentence deleted |
