# Supplementary material for: Correlation between quality of life of cardiac patients and caregiver burden
Source: PLoS One. 2020 Aug 3;15(8):e0237099. doi: 10.1371/journal.pone.0237099 (PMC7398537; doi:10.1371/journal.pone.0237099)
Supplement: Attachment — Submitted filename: Response to Reviewers.docx [file pone.0237099.s003.docx]

**Response to Reviewers**

|  | **Editor comments** | **response** |
| --- | --- | --- |
| 1- | Given this is a cross-sectional study, I think the term 'predict/predictor/prediction' should be replaced with 'correlation' or 'association' throughout, including the title. | Changed to correlation including the title |
| 2- | Please delete the sentence at the end of the Introduction, which is repetitious and poorly constructed: 'Hence the novelty of this study is to examine the QoL of cardiac patients with the caregiver burden'. | deleted |
| 3- | Please rephrase the objectives to read: 'The purpose of this paper was to assess the QoL of cardiac patients and its association with caregivers’ burden alongside other variables'. | rephrased |
| 4- | Please remove reference to anonymity as, technically, the data were re-identifiable. | removed |
| 5- | Please change to: 'Cronbach’s alpha reliability was previously found to be between 0.80 and 0.85'. | changed |
| 6- | Please change to: "Cronbach’s alphas have previously measured between .84 and .98 for the original QLI-Cardiac 4 (total scale)' | changed |
| 7- | Please change data to be plural rather than singular throughout (e.g. 'were' not 'was' analysed). | changed |
| 8- | Please change 'our norms and culture'' to 'Jordanian norms and culture'. | changed |
| 9- | Please explain further the following sentence: 'Therefore, the caregiver burden was moderate in this study as the burden of providing care for the patient after discharge primarily lies with family members, especially if the patient with cardiac disease is a parent' | Changed “Therefore, the level of burden in the current study was moderate, not high. The possible explanation is that family members share the burden of providing care in particulate when the affected person is one of the parents (24). That is, sharing this responsibility reduce the burden on the caregivers” |
| 10- | New paragraph commencing with 'Day-to-day life with patients with cardiac disease ...' - does the first sentence imply that there may be a causal relationship between caregiver burden and patient QOL? If so, given this is cross-sectional data, an alternative interpretation might also need to be offered that sees the relationship the other way around or merely associative and not causal. I don't see how the other sentences in this paragraph refer to the correlation between patient QOL and CG burden? | Modified by adding a sentence “The relationship between the QoL and caregiver burden are going in both directions. “ |
| 11- | The limitation on convenience sampling and generalisability should be separated from the one on cross-sectional data and causal inference in two different sentences. | Had been separated |
| 12- | Please remove the first sentence which does not relate to findings from the study and replace with one that restates that this study found a correlation between patient QOL and caregiver burden. | Removed and replaced with editor suggestion. |
| 13- | All previous comments from reviewers had been adjusted | |
