# Supplementary material for: Correlation between quality of life of cardiac patients and caregiver burden
Source: PLoS One. 2020 Aug 3;15(8):e0237099. doi: 10.1371/journal.pone.0237099 (PMC7398537; doi:10.1371/journal.pone.0237099)
Supplement: Attachment — Submitted filename: Response to Reviewers.docx [file pone.0237099.s004.docx]

'Response to Reviewers'

Editor comment “the insertion of "the relationship between the QoL and caregiver burden are going in both directions" into the Discussion cannot be justified based on the available data, nor does it adequately introduce the remainder of the paragraph, which seems to only focus on one direction and also needs editing for English. I suggest replacing this paragraph with the following:

While the cross-sectional nature of the current study does not allow inferences to be drawn about the relationships between patient QoL and caregiver burden, we can speculate about possible explanations for the correlation. Day-to-day life with patients with cardiac disease often brings new physical, psychosocial, and financial challenges for families. To address these challenges caregivers may exert more efforts to keep their patients in good QoL, experiencing hardships and stress in the process (35). This may be heightened by the threat of losing a partner and/or the added burden of caring for someone who was previously able to contribute to household responsibilities.”

- What you request to do had been done.
